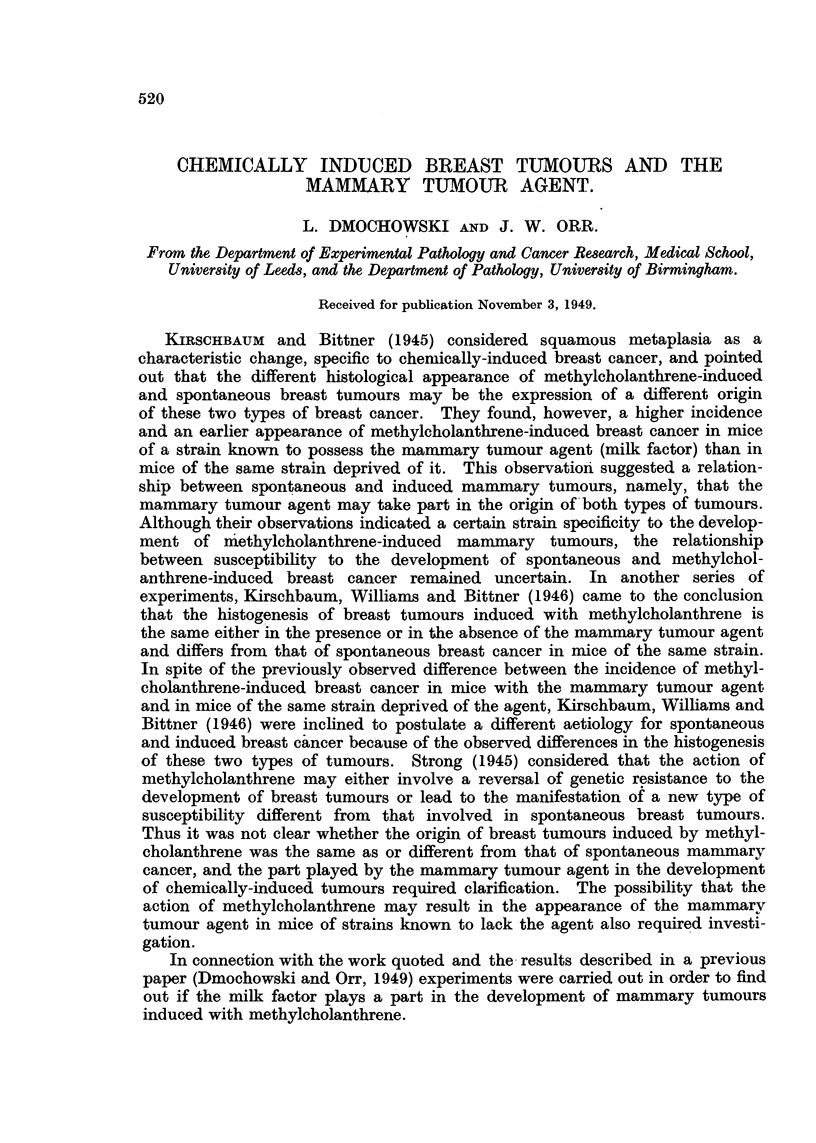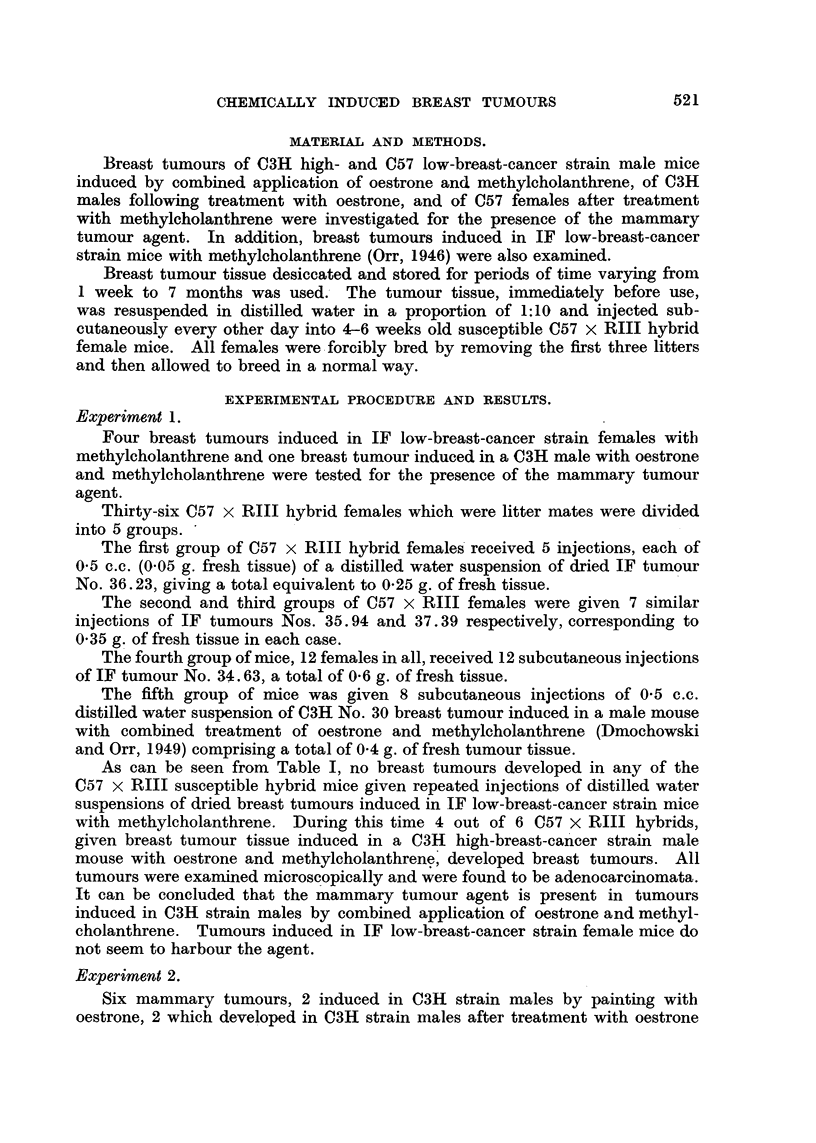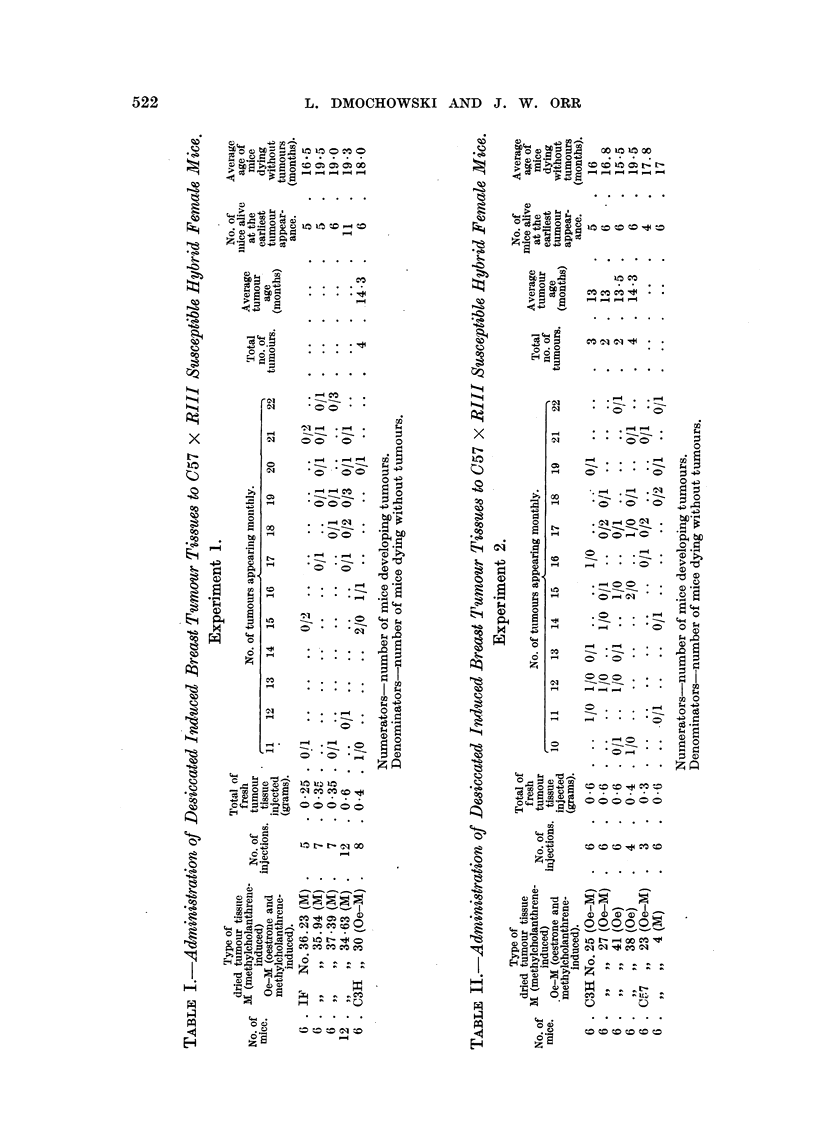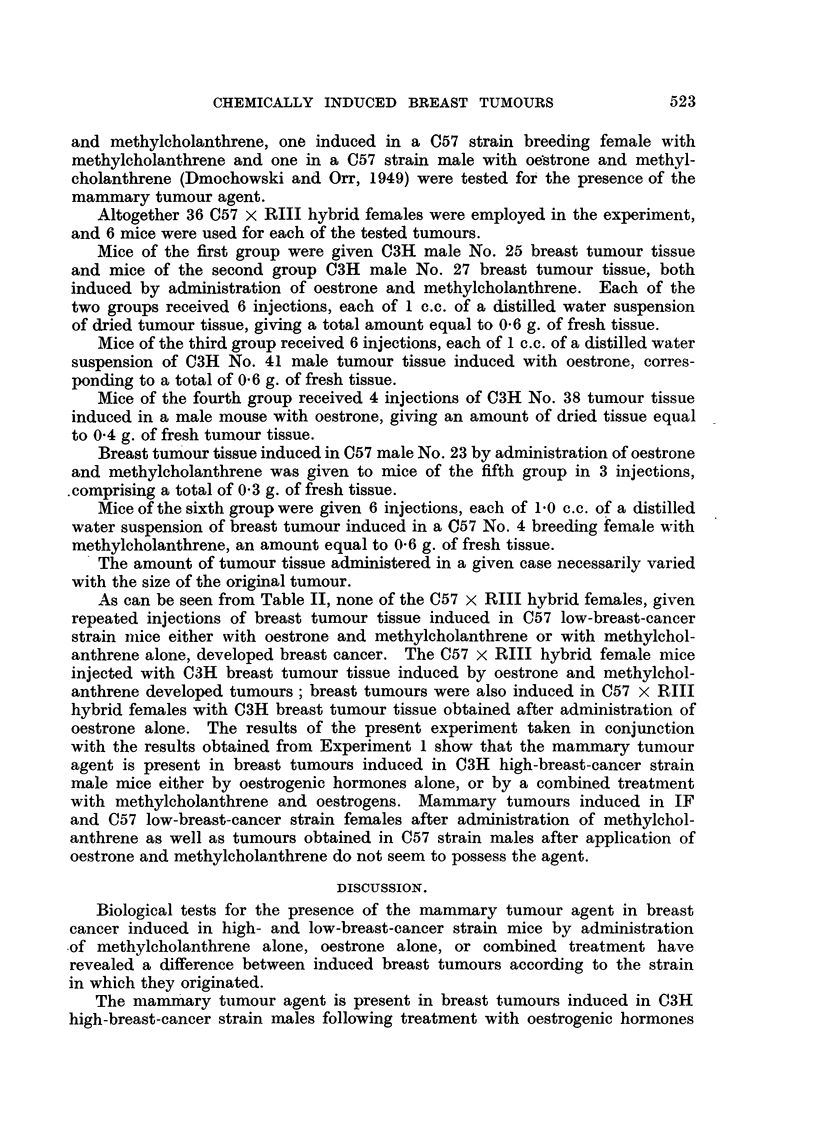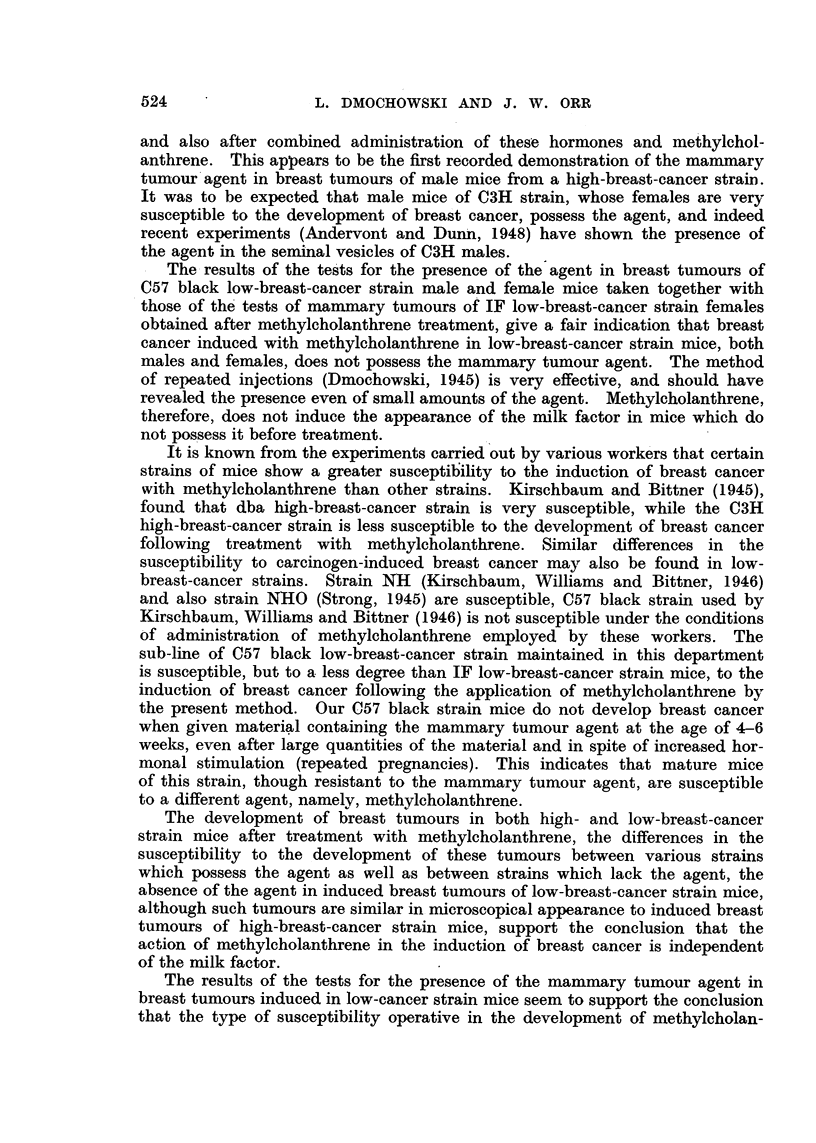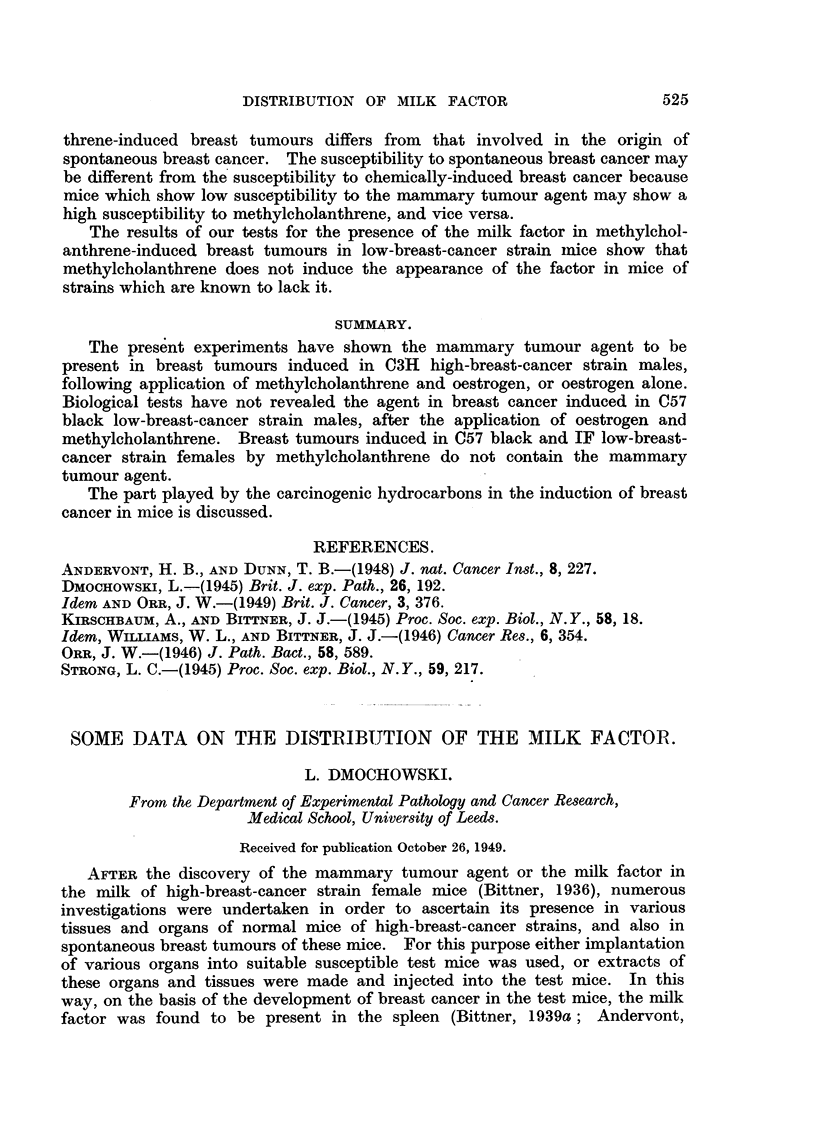# Chemically Induced Breast Tumours and the Mammary Tumour Agent

**DOI:** 10.1038/bjc.1949.55

**Published:** 1949-12

**Authors:** L. Dmochowski, J. W. Orr


### CHEMICALLY INDUCED BREAST TUMOURS AND THE MAMMARY TUMOUR AGENT.

L. DMOCHOWSKI AND J. W. ORR.

*From the Department of Experimental Pathology and Cancer Research, Medical School, University of Leeds, and the Department of Pathology, University of Birmingham.*

Received for publication November 3, 1949.

KIRSCHBAUM and Bittner (1945) considered squamous metaplasia as a characteristic change, specific to chemically-induced breast cancer, and pointed out that the different histological appearance of methylcholanthrene-induced and spontaneous breast tumours may be the expression of a different origin of these two types of breast cancer. They found, however, a higher incidence and an earlier appearance of methylcholanthrene-induced breast cancer in mice of a strain known to possess the mammary tumour agent (milk factor) than in mice of the same strain deprived of it. This observation suggested a relationship between spontaneous and induced mammary tumours, namely, that the mammary tumour agent may take part in the origin of both types of tumours. Although their observations indicated a certain strain specificity to the development of methylcholanthrene-induced mammary tumours, the relationship between susceptibility to the development of spontaneous and methylcholanthrene-induced breast cancer remained uncertain. In another series of experiments, Kirschbaum, Williams and Bittner (1946) came to the conclusion that the histogenesis of breast tumours induced with methylcholanthrene is the same either in the presence or in the absence of the mammary tumour agent and differs from that of spontaneous breast cancer in mice of the same strain. In spite of the previously observed difference between the incidence of methylcholanthrene-induced breast cancer in mice with the mammary tumour agent and in mice of the same strain deprived of the agent, Kirschbaum, Williams and Bittner (1946) were inclined to postulate a different aetiology for spontaneous and induced breast cancer because of the observed differences in the histogenesis of these two types of tumours. Strong (1945) considered that the action of methylcholanthrene may either involve a reversal of genetic resistance to the development of breast tumours or lead to the manifestation of a new type of susceptibility different from that involved in spontaneous breast tumours. Thus it was not clear whether the origin of breast tumours induced by methylcholanthrene was the same as or different from that of spontaneous mammary cancer, and the part played by the mammary tumour agent in the development of chemically-induced tumours required clarification. The possibility that the action of methylcholanthrene may result in the appearance of the mammary tumour agent in mice of strains known to lack the agent also required investigation.

In connection with the work quoted and the results described in a previous paper (Dmochowski and Orr, 1949) experiments were carried out in order to find out if the milk factor plays a part in the development of mammary tumours induced with methylcholanthrene.

### MATERIAL AND METHODS.

Breast tumours of C3H high- and C57 low-breast-cancer strain male mice induced by combined application of oestrone and methylcholanthrene, of C3H males following treatment with oestrone, and of C57 females after treatment with methylcholanthrene were investigated for the presence of the mammary tumour agent. In addition, breast tumours induced in IF low-breast-cancer strain mice with methylcholanthrene (Orr, 1946) were also examined.

Breast tumour tissue desiccated and stored for periods of time varying from 1 week to 7 months was used. The tumour tissue, immediately before use, was resuspended in distilled water in a proportion of 1:10 and injected subcutaneously every other day into 4–6 weeks old susceptible C57 × RIII hybrid female mice. All females were forcibly bred by removing the first three litters and then allowed to breed in a normal way.

### EXPERIMENTAL PROCEDURE AND RESULTS.

*Experiment* 1.

Four breast tumours induced in IF low-breast-cancer strain females with methylcholanthrene and one breast tumour induced in a C3H male with oestrone and methylcholanthrene were tested for the presence of the mammary tumour agent.

Thirty-six C57 × RIII hybrid females which were litter mates were divided into 5 groups.

The first group of C57 × RIII hybrid females received 5 injections, each of 0·5 c.c. (0·05 g. fresh tissue) of a distilled water suspension of dried IF tumour No. 36.23, giving a total equivalent to 0·25 g. of fresh tissue.

The second and third groups of C57 × RIII females were given 7 similar injections of IF tumours Nos. 35.94 and 37.39 respectively, corresponding to 0·35 g. of fresh tissue in each case.

The fourth group of mice, 12 females in all, received 12 subcutaneous injections of IF tumour No. 34.63, a total of 0·6 g. of fresh tissue.

The fifth group of mice was given 8 subcutaneous injections of 0·5 c.c. distilled water suspension of C3H No. 30 breast tumour induced in a male mouse with combined treatment of oestrone and methylcholanthrene (Dmochowski and Orr, 1949) comprising a total of 0·4 g. of fresh tumour tissue.

As can be seen from Table I, no breast tumours developed in any of the C57 × RIII susceptible hybrid mice given repeated injections of distilled water suspensions of dried breast tumours induced in IF low-breast-cancer strain mice with methylcholanthrene. During this time 4 out of 6 C57 × RIII hybrids, given breast tumour tissue induced in a C3H high-breast-cancer strain male mouse with oestrone and methylcholanthrene, developed breast tumours. All tumours were examined microscopically and were found to be adenocarcinomata. It can be concluded that the mammary tumour agent is present in tumours induced in C3H strain males by combined application of oestrone and methylcholanthrene. Tumours induced in IF low-breast-cancer strain female mice do not seem to harbour the agent.

*Experiment* 2.

Six mammary tumours, 2 induced in C3H strain males by painting with oestrone, 2 which developed in C3H strain males after treatment with oestrone

TABLE I.—*Administration of Desiccated Induced Breast Tumour Tissues to C57 × RIII Susceptible Hybrid Female Mice.*

Experiment 1.

and methylcholanthrene, one induced in a C57 strain breeding female with methylcholanthrene and one in a C57 strain male with oestrone and methylcholanthrene (Dmochowski and Orr, 1949) were tested for the presence of the mammary tumour agent.

Altogether 36 C57 × RIII hybrid females were employed in the experiment, and 6 mice were used for each of the tested tumours.

Mice of the first group were given C3H male No. 25 breast tumour tissue and mice of the second group C3H male No. 27 breast tumour tissue, both induced by administration of oestrone and methylcholanthrene. Each of the two groups received 6 injections, each of 1 c.c. of a distilled water suspension of dried tumour tissue, giving a total amount equal to 0·6 g. of fresh tissue.

Mice of the third group received 6 injections, each of 1 c.c. of a distilled water suspension of C3H No. 41 male tumour tissue induced with oestrone, corresponding to a total of 0·6 g. of fresh tissue.

Mice of the fourth group received 4 injections of C3H No. 38 tumour tissue induced in a male mouse with oestrone, giving an amount of dried tissue equal to 0·4 g. of fresh tumour tissue.

Breast tumour tissue induced in C57 male No. 23 by administration of oestrone and methylcholanthrene was given to mice of the fifth group in 3 injections, comprising a total of 0·3 g. of fresh tissue.

Mice of the sixth group were given 6 injections, each of 1·0 c.c. of a distilled water suspension of breast tumour induced in a C57 No. 4 breeding female with methylcholanthrene, an amount equal to 0·6 g. of fresh tissue.

The amount of tumour tissue administered in a given case necessarily varied with the size of the original tumour.

As can be seen from Table II, none of the C57 × RIII hybrid females, given repeated injections of breast tumour tissue induced in C57 low-breast-cancer strain mice either with oestrone and methylcholanthrene or with methylcholanthrene alone, developed breast cancer. The C57 × RIII hybrid female mice injected with C3H breast tumour tissue induced by oestrone and methylcholanthrene developed tumours ; breast tumours were also induced in C57 × RIII hybrid females with C3H breast tumour tissue obtained after administration of oestrone alone. The results of the present experiment taken in conjunction with the results obtained from Experiment 1 show that the mammary tumour agent is present in breast tumours induced in C3H high-breast-cancer strain male mice either by oestrogenic hormones alone, or by a combined treatment with methylcholanthrene and oestrogens. Mammary tumours induced in IF and C57 low-breast-cancer strain females after administration of methylcholanthrene as well as tumours obtained in C57 strain males after application of oestrone and methylcholanthrene do not seem to possess the agent.

### DISCUSSION.

Biological tests for the presence of the mammary tumour agent in breast cancer induced in high- and low-breast-cancer strain mice by administration of methylcholanthrene alone, oestrone alone, or combined treatment have revealed a difference between induced breast tumours according to the strain in which they originated.

The mammary tumour agent is present in breast tumours induced in C3H high-breast-cancer strain males following treatment with oestrogenic hormones and also after combined administration of these hormones and methylcholanthrene. This appears to be the first recorded demonstration of the mammary tumour agent in breast tumours of male mice from a high-breast-cancer strain. It was to be expected that male mice of C3H strain, whose females are very susceptible to the development of breast cancer, possess the agent, and indeed recent experiments (Andervont and Dunn, 1948) have shown the presence of the agent in the seminal vesicles of C3H males.

The results of the tests for the presence of the agent in breast tumours of C57 black low-breast-cancer strain male and female mice taken together with those of the tests of mammary tumours of IF low-breast-cancer strain females obtained after methylcholanthrene treatment, give a fair indication that breast cancer induced with methylcholanthrene in low-breast-cancer strain mice, both males and females, does not possess the mammary tumour agent. The method of repeated injections (Dmochowski, 1945) is very effective, and should have revealed the presence even of small amounts of the agent. Methylcholanthrene, therefore, does not induce the appearance of the milk factor in mice which do not possess it before treatment.

It is known from the experiments carried out by various workers that certain strains of mice show a greater susceptibility to the induction of breast cancer with methylcholanthrene than other strains. Kirschbaum and Bittner (1945), found that dba high-breast-cancer strain is very susceptible, while the C3H high-breast-cancer strain is less susceptible to the development of breast cancer following treatment with methylcholanthrene. Similar differences in the susceptibility to carcinogen-induced breast cancer may also be found in low-breast-cancer strains. Strain NH (Kirschbaum, Williams and Bittner, 1946) and also strain NHO (Strong, 1945) are susceptible, C57 black strain used by Kirschbaum, Williams and Bittner (1946) is not susceptible under the conditions of administration of methylcholanthrene employed by these workers. The sub-line of C57 black low-breast-cancer strain maintained in this department is susceptible, but to a less degree than IF low-breast-cancer strain mice, to the induction of breast cancer following the application of methylcholanthrene by the present method. Our C57 black strain mice do not develop breast cancer when given material containing the mammary tumour agent at the age of 4–6 weeks, even after large quantities of the material and in spite of increased hormonal stimulation (repeated pregnancies). This indicates that mature mice of this strain, though resistant to the mammary tumour agent, are susceptible to a different agent, namely, methylcholanthrene.

The development of breast tumours in both high- and low-breast-cancer strain mice after treatment with methylcholanthrene, the differences in the susceptibility to the development of these tumours between various strains which possess the agent as well as between strains which lack the agent, the absence of the agent in induced breast tumours of low-breast-cancer strain mice, although such tumours are similar in microscopical appearance to induced breast tumours of high-breast-cancer strain mice, support the conclusion that the action of methylcholanthrene in the induction of breast cancer is independent of the milk factor.

The results of the tests for the presence of the mammary tumour agent in breast tumours induced in low-cancer strain mice seem to support the conclusion that the type of susceptibility operative in the development of methylcholanthrene-induced breast tumours differs from that involved in the origin of spontaneous breast cancer. The susceptibility to spontaneous breast cancer may be different from the susceptibility to chemically-induced breast cancer because mice which show low susceptibility to the mammary tumour agent may show a high susceptibility to methylcholanthrene, and vice versa.

The results of our tests for the presence of the milk factor in methylcholanthrene-induced breast tumours in low-breast-cancer strain mice show that methylcholanthrene does not induce the appearance of the factor in mice of strains which are known to lack it.

### SUMMARY.

The present experiments have shown the mammary tumour agent to be present in breast tumours induced in C3H high-breast-cancer strain males, following application of methylcholanthrene and oestrogen, or oestrogen alone. Biological tests have not revealed the agent in breast cancer induced in C57 black low-breast-cancer strain males, after the application of oestrogen and methylcholanthrene. Breast tumours induced in C57 black and IF low-breast-cancer strain females by methylcholanthrene do not contain the mammary tumour agent.

The part played by the carcinogenic hydrocarbons in the induction of breast cancer in mice is discussed.

### REFERENCES.

ANDERVONT, H. B., AND DUNN, T. B.—(1948) *J. nat. Cancer Inst.*, **8**, 227.
DMOCHOWSKI, L.—(1945) *Brit. J. exp. Path.*, **26**, 192.
*Idem* AND ORR, J. W.—(1949) *Brit. J. Cancer*, **3**, 376.
KIRSCHBAUM, A., AND BITTNER, J. J.—(1945) *Proc. Soc. exp. Biol., N.Y.*, **58**, 18.
*Idem*, WILLIAMS, W. L., AND BITTNER, J. J.—(1946) *Cancer Res.*, **6**, 354.
ORR, J. W.—(1946) *J. Path. Bact.*, **58**, 589.
STRONG, L. C.—(1945) *Proc. Soc. exp. Biol., N.Y.*, **59**, 217.

### SOME DATA ON THE DISTRIBUTION OF THE MILK FACTOR.

L. DMOCHOWSKI.